# Clinicians’ confidence in synthetic neuroimaging: a survey from the EANS emerging Technologies and Innovations in Neurosurgery (ETIN) taskforce

**DOI:** 10.1016/j.bas.2026.107488

**Published:** 2026-10-22

**Authors:** Michelle D. Poelman, Massimo Bottini, Olivier Zanier, Istiak Khan, Daniel De Wilde, Stefanos Voglis, Adrian Elmi-Terander, Victor Gabriel El-Hajj, Nikolay Gabrovsky, Alessandro Boaro, Stefan Motov, Eric Suero Molina, Seung-Jun Ryu, Antonio Di Ieva, Marisa Gandia González, Luca Regli, Carlo Serra, Marc L. Schröder, Victor E. Staartjes

**Affiliations:** a Department of Neurosurgery, Park Medical Center, Rotterdam, the Netherlands; b Machine Intelligence in Clinical Neuroscience & Microsurgical Neuroanatomy (MICN) Laboratory, Department of Neurosurgery, Clinical Neuroscience Center, University Hospital Zurich, University of Zurich, Switzerland; c Capio Spine Center Stockholm, Löwenströmska Hospital, Upplands-Väsby, Sweden; d Department of Clinical Neuroscience, Karolinska Institutet, Stockholm, Sweden; e European Association of Neurosurgical Societies (EANS) Emerging Technologies and Innovations (ETIN) Taskforce, Brussels, Belgium; f Department of Neurosurgery, University Hospital Münster, Münster, Germany; g Macquarie Medical School, Macquarie University, Sydney, Australia; h Department of Neurosurgery, Daejeon Eulji University Hospital, Eulji University Medical School, Daejeon, South Korea; i Department of Neurosurgery, Macquarie University Hospital, Sydney, Australia; j Department of Neurosurgery, La Paz University Hospital, Madrid, Spain

**Keywords:** Confidence, Machine learning, Neurosurgery, Survey, Synthetic CT, Synthetic imaging

## Abstract

**Introduction:**

Neurosurgery relies heavily on imaging for diagnostic, planning, intra-operative decision-making, and image-guided interventions. Machine learning (ML) enables synthetic imaging, such as synthetic CT (sCT) generated from MRI or biplanar X-rays, potentially reducing radiation exposure and improving imaging access. However, clinicians’ confidence in synthetic imaging remains poorly understood.

**Research question:**

This study assessed respondents attitudes towards synthetic neuroimaging.

**Material and methods:**

An international cross-sectional survey was distributed via the European Association of Neurosurgical Societies (EANS) and a global neurosurgeon database. Nineteen questions covered demographics, general attitudes, and two algorithms: MRI-based sCT, and biplanar X-ray-based sCT. Confidence was reported using a 5-point Likert scale (1 = not confident, 5 = completely confident). Responses were summarized using medians, interquartile ranges (IQR), counts, and percentages.

**Results:**

Seventy-two respondents completed the survey; 61% were board-certified neurosurgeons, and 76% worked in academic hospitals. Confidence varied across the two clinical scenarios. Confidence for MRI-based spinal decision-making was moderate (median 3, IQR 3-4), whereas confidence in X-ray-based sCT for cranial applications was lower (median 2, IQR 2-3). Clinical and external validation were frequently selected as factors supporting confidence, whereas hallucinations and lack of validation limited confidence.

**Discussion and conclusion:**

Confidence in synthetic neuroimaging varied across clinical scenarios. Differences may reflect clinical context, perceived consequences of error, input modality, and information provided in each scenario. While synthetic imaging may improve access, reduce radiation exposure, and support decision-making, adoption requires robust, application-specific validation. These findings guide future implementation of synthetic imaging in neurosurgery.

## Highlights

• Survey of clinicians' confidence in synthetic neuroimaging.
• Clinical validation was the most frequently reported trust-factor.
• Confidence differed between the two clinical scenarios.
• Potential benefits include improved access and reduced radiation exposure.
• Findings inform future clinical evaluation and implementation.

## 1 Introduction

Neurosurgical decision-making highly depends on imaging-derived information for diagnosis, operative planning, and intraoperative neuronavigation. These decisions are not purely technical but rely on the clinician's interpretative expertise for high-risk interventions. Recent advances in machine learning (ML) have enabled the generation of synthetic medical images through image modality conversion, e.g. transforming Magnetic Resonance Imaging (MRI) into synthetic Computed Tomography (sCT), or generating sCT from multiple X-ray projections (Alakmeh et al., 2025). These methods are currently being developed and validated and have clear potential to aid neurosurgical patients. Studies suggest that sCT can provide visualization of osseous anatomy (Tan et al., 2024; Jiang et al., 2025; Shilo et al., 2025) while reducing radiation exposure, streamlining surgical workflows (Jiang et al., 2025; Shilo et al., 2025). By eliminating the need for a separate CT scan, sCT may also reduce costs and improve accessibility, which facilitates surgery in settings with limited CT availability. Despite rapid technological progress, clinical adoption remains subject to challenges involving standardization and validation (De Wilde et al., 2025).

There is a fundamental difference between how conventional images and synthetic images are generated. In conventional imaging, voxel intensities are reconstructed from direct physical measurements of X-ray attenuation (Staartjes et al., 2026). In synthetic imaging, some voxels are constructed by an algorithm from lower-dimensional input. Therefore, depending on the input data, model architecture, training distribution, and clinical adaptation, synthetic imaging models are susceptible to reconstruction errors (Alakmeh et al., 2025). For example, model overfitting, hallucinations (i.e., inaccurate anatomical representations of structures), or extrapolations beyond the information contained in the input data may result in incorrect representations of the patient's anatomy in the synthetic image. These potential failure modes highlight the importance of evaluating synthetic imaging not only through technical image-quality metrics, but also through application-specific clinical validation.

Nevertheless, even if in-silico performance is robust, technical performance alone does not determine whether a synthetic imaging method will be accepted for a specific task. Its clinical adaptation ultimately depends on neurosurgeons' confidence in synthetic imaging. The ultimate challenge for a model to be integrated into clinical practice is to build sufficient confidence among clinicians and patients. However, evidence regarding clinicians’ confidence in using synthetic images for specific diagnostic and therapeutic decisions remains limited. This includes decisions such as confirming a diagnosis, excluding a condition, proceeding with surgery without additional imaging, or discharging a patient based on synthetic imaging.

This study therefore aimed to assess current attitudes towards synthetic neuroimaging among respondents recruited through neurosurgical professional networks, using two predefined clinical scenarios relevant to neurosurgical practice. We evaluated responses to two clinical scenarios involving MRI-based sCT for spinal applications and biplanar X-ray-based sCT for cranial applications.

## 2 Methods

### 2.1 Study design and participants

A cross-sectional international survey was conducted to assess confidence in and attitudes towards synthetic neuroimaging. The survey was distributed through the European Association of Neurosurgical Societies (EANS) in July and August of 2025. EANS, which has around 2700 members, sent our survey via their monthly newsletter and official webpage on 24 July 2025. In addition, a global neurosurgeon database containing 8971 e-mail addresses from hospital website data was used, of which 7173 were confirmed as active at the time of distribution. This survey was supported by the EANS Emerging Technologies and Innovations in Neurosurgery (ETIN) Taskforce.

The survey was open to respondents reached through stated distribution channels and respondents were asked to report their professional background. Respondents included board-certified neurosurgeons, neurosurgical trainees, radiologists, medical researchers, researchers, medical students or other professionals. The study population therefore consisted of respondents with varying levels of clinical and neurosurgical experience.

The survey was developed and distributed using Qualtrics survey software (Qualtrics, Provo, UT). Participation was voluntary, all answers were collected anonymously, and no incentives were provided. Respondents were informed about the purpose of the study before starting the questionnaire. Consent was implied by voluntary completion of the survey. To ensure that answers were obtained from unique site visitors, each email address was only allowed to complete the survey once. This was not possible to prevent for respondents who accessed the survey via an anonymous link. Survey design and reporting were guided by the Checklist for Reporting Results of Internet E-Surveys (CHERRIES) guidelines, where applicable (Eysenbach, 2004).

The survey consisted of 19 questions, of which 6 collected basic demographic information to characterize the survey population. The remaining questions can be categorized into three domains: 1) Attitudes towards synthetic imaging for diagnostic purposes, 2) Attitudes towards synthetic imaging for therapeutic purposes, and 3) General attitudes towards synthetic imaging. Questions were presented in this fixed order. Two specific algorithms were presented, including concise explanations of the algorithms and relevant. An example of the synthetic image alongside a reference conventional CT image was provided. The survey then asked respondents to rate their confidence in specific diagnostic or therapeutic decisions. No formal sample size calculation was performed, sample size was determined by the number of respondents reached through stated distribution channels.

### 2.2 Algorithm 1 – synthetic CT from MRI

Algorithm 1 (Fig. 1) represented a previously published BoneMRI approach for generating sCT images of the lumbar spine from MRI. The method used an additional 3D radiofrequency-spoiled T1-weighted multiple gradient-echo (MGE) MRI sequence, intended to provide information related to osseous anatomy. Then, a convolutional neural network (CNN), used to generate voxel-wise CT-like radiodensity estimates from MRI data, transformed the MRI data into CT (Staartjes et al., 2021). The algorithm has been trained on paired MRI-CT datasets and has subsequently been evaluated in independent clinical cohorts, including comparing sCT with low-dose CT (Staartjes et al., 2021; Willesen et al., 2024). For the present study, a representative MRI-sCT-CT image pair from this approach was presented to respondents as an example of MRI-based sCT for spinal applications.

**Fig. 1:**
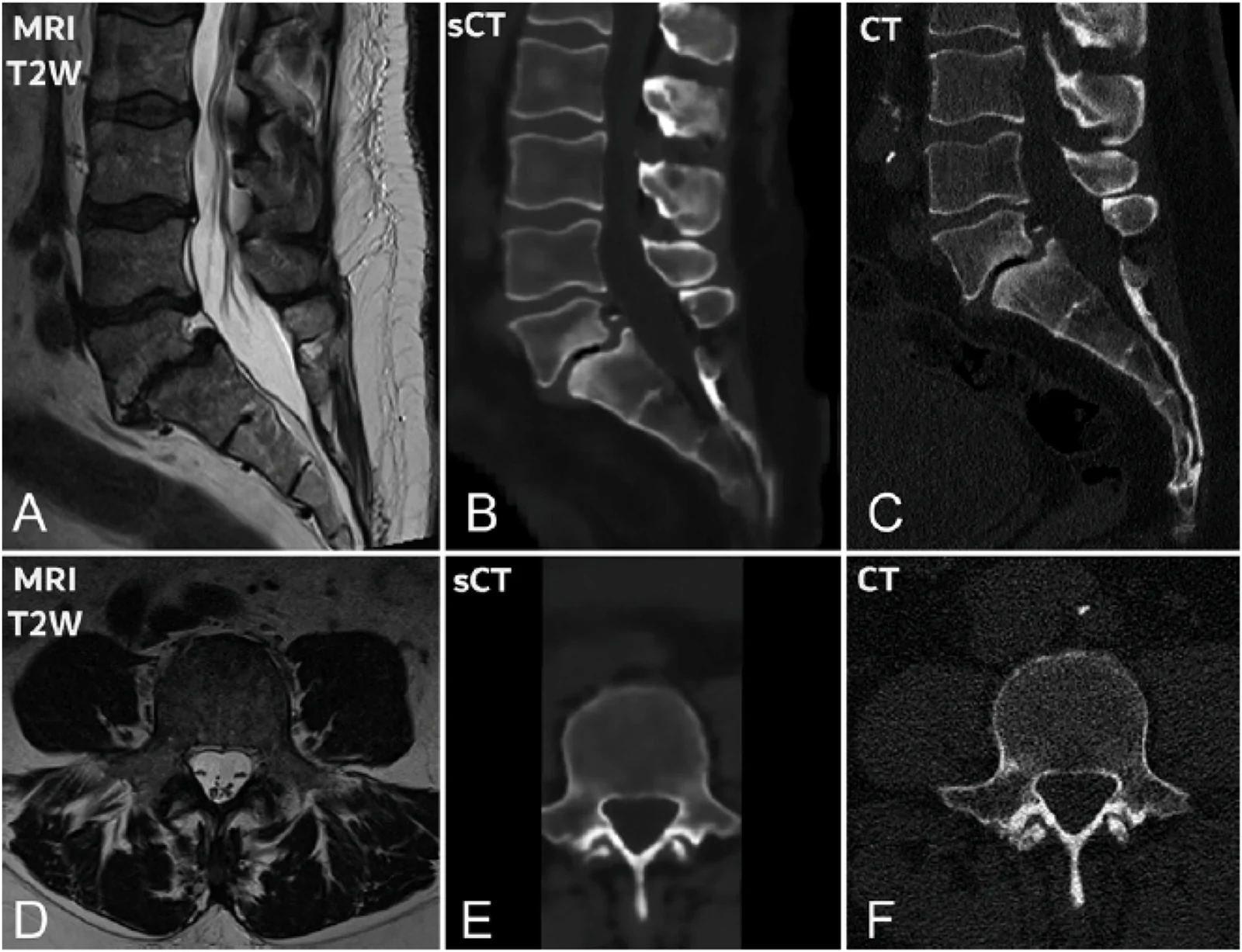
Spinal scenario. MRI input (A,D), MRI-derived synthetic CT (B,E), and corresponding reference conventional CT (C,F) for spinal application.

### 2.3 Algorithm 2 – synthetic CT of biplanar X-ray

Algorithm 2 (Fig. 2) represented a previously published generative adversarial network (GAN) for generating a tomography-like image from anteroposterior and lateral X-rays of the skull and brain (TomoRay Cranial) (Zanier et al., 2026). The GAN architecture was based on the X2CT-GAN framework, which was selected because of its established application to 2D-to-3D CT reconstruction from radiographic projections (Zanier et al., 2026). For the present study, a representative biplanar X-ray-sCT image pair from this approach was presented to respondents as an example of X-ray-based sCT for cranial applications.

**Fig. 2:**
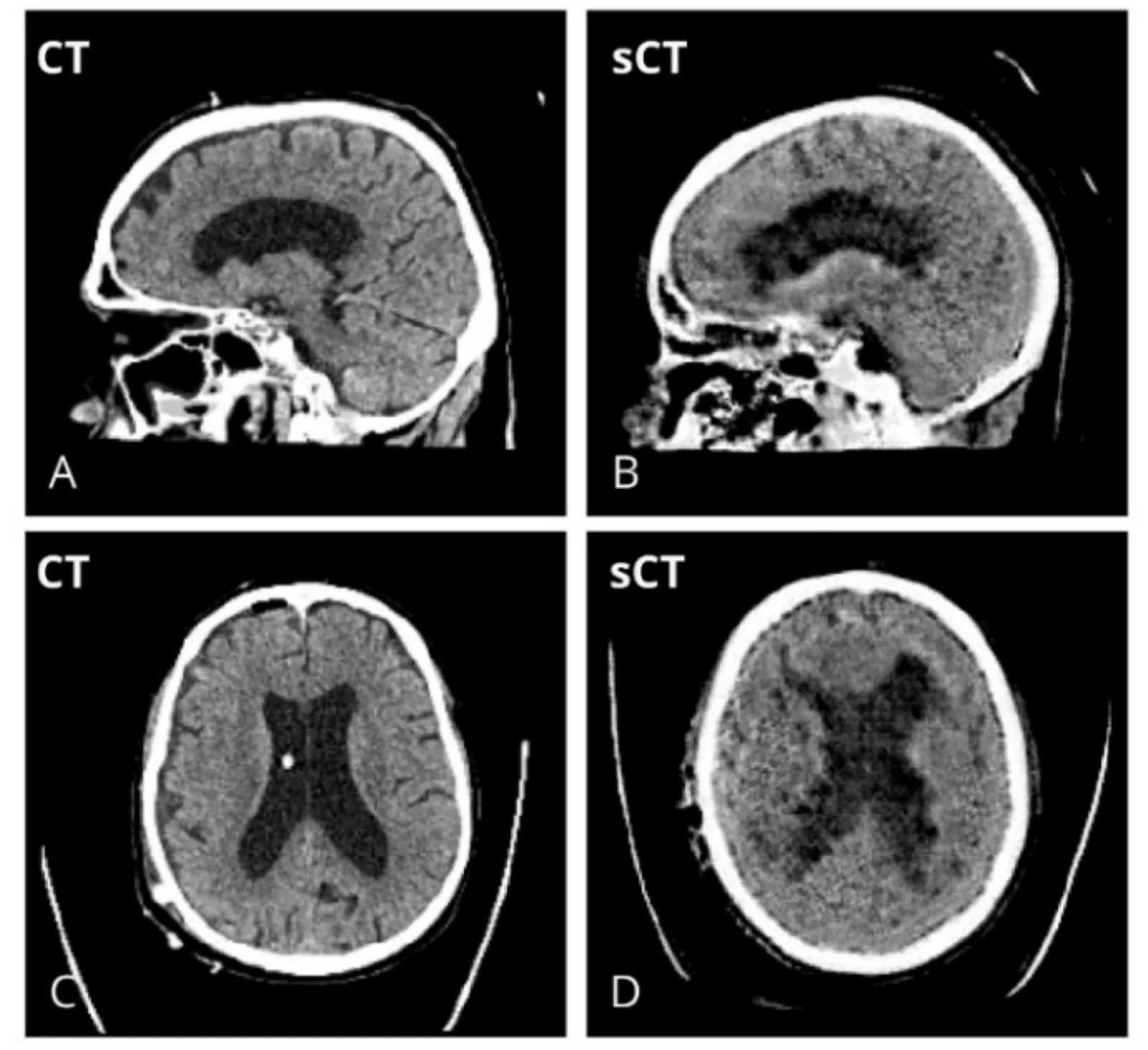
Cranial scenario. Reference conventional CT (A,C) and synthetic CT (B,D) generated from biplanar X-ray projections for cranial application.

Algorithm-specific questions are presented in Table 1, Table 2. Questions addressing general attitudes towards synthetic imaging can be found in Table 3, and a complete overview of all questions and response options is provided in (**Supplementary**) Table 7.

**Table 1:** Algorithm 1.

**Algorithm 1: Synthetic CT from MRI** This algorithm is based on an MRI sequence that adds a couple of minutes to a normal MRI and teases out some information about the bone structure. Then, a convolutional neural network (CNN) transforms the MRI to a synthetic CT (sCT). The algorithm has been trained on hundreds of paired data sets and it has been externally validated. The same technique has also been successfully applied to other joints/bones. The training data set included several patients with pars fractures.	
How confident are you in determining (ruling in) the presence of a pars fracture based solely on the synthetic image?	Confidence 1-5
If the sCT shows signs of a pars fracture, how necessary would you deem additional conventional imaging to confirm the diagnosis?	Necessary 1-5
If there were an indication for surgery, how confident would you be in undergoing surgery without additional conventional imaging?	Confidence 1-5
How confident would you be in placing lumbar pedicle screws using a navigation system based solely on the synthetic images (sCT) without additional imaging?	Confidence 1-5

**Table 2:** Algorithm 2.

**Algorithm 2: Synthetic CT of biplanar X-ray** This algorithm is based on a generative adversarial network (GAN) that can synthesize a tomography-like image from a.p. and lateral X-rays of the skull and brain. The algorithm has been developed on hundreds of paired data sets. It has been externally validated. During external validation, the algorithm has been able to pick up most but not all of the hydrocephalus cases. The training data set included several patients with hydrocephalus.	
How confident are you in diagnosing or excluding a hydrocephalus based solely on the synthetic image?	Confidence 1-5
If the sCT does not show a hydrocephalus, how necessary do you deem additional conventional imaging to safely exclude (ruling out) a possible hydrocephalus?	Necessary 1-5
How confident would you be in discharging the patient home based only on the synthetic image?	Confidence 1-5
How confident would you be in placing a navigated external ventricular drain based on a navigation system based only on the synthetic imaging?	Confidence 1-5

**Table 3:** General attitude.

General attitude	
In general, to what extent would you trust synthetic images (images that are partially generated by algorithms, rather than every voxel/pixel being based on a physical measurement) for **making a diagnosis**?	Trust 1-5
In general, to what extent would you trust synthetic images (images that are partially generated by algorithms, rather than every voxel/pixel being based on a physical measurement) for **guiding interventions**?	Trust 1-5
Factors important to you in building confidence in synthetic imaging applications	1. Variability of diseases present in the training data 2. Size of the training data set 3. Presence of external validation 4. Presence of clinical validation studies for specific clinical queries (e.g. rule-in of fractures, rule-out of bleeding) 5. Peer-reviewed publications 6. Licensing by a known MedTech brand
Factors important to you in limiting confidence in synthetic imaging applications:	1. Hallucinations made by generative algorithms 2. Poor or non-existing external validation 3. No clinical testing for specific clinical queries 4. Algorithms provided for free without involvement of a known MedTech brand
Where do you see potential benefits for synthetic imaging (e.g. the possibility to generate a moderate-quality synthetic CT from two conventional X-rays)?	1. Low-middle income or rural areas without easy access to/with very long waiting lists for tomographic imaging 2. Severely ill patients on the Neuro-ICU who would otherwise require transfer to radiology 3. Pre-hospital situations (i.e. rough initial diagnostics/triage) 4. The neurosurgical operating room/guidance of interventions 5. Replacing conventional imaging methods for certain clinical queries 6. Reducing radiation exposure in patients requiring frequent imaging

### 2.4 Data analysis

Categorical data were summarized using numbers and percentages. Ordinal data derived from Likert-scales were summarized using medians and interquartile ranges (IQRs), together with the frequency and percentage of responses in each response category where applicable (Sullivan and Artino, 2013). Confidence intervals (CI) were also reported where necessary. Respondents reported their confidence using a 5-point Likert Scale (ranging from 1 = not confident at all to 5 = completely confident). Questions concerning the perceived necessity of additional imaging used a 5-point scale (ranging from 1 = not necessary at all to 5 = absolutely necessary). General trust-related questions used a 5-point scale ranging from 1 (no trust at all) to 5 (complete trust).

All completed responses were included in the primary descriptive analysis, irrespective of professional background. Therefore, the reported confidence and trust ratings represent the responses of all survey participants and should not be interpreted as estimates of neurosurgeon's attitudes exclusively.

## 3 Results

### 3.1 Response rate and characteristics

A total of 132 people started the survey and a total of 72 (55%) respondents finished the survey and completed all questions. Of these, 44 (61%) identified as board-certified neurosurgeons and 14 (19%) as neurosurgical trainees. The remaining respondents included one radiologist, four medical researchers, three researchers and six medical students. Thus, 58 respondents (81%) were either board-certified neurosurgeons or neurosurgical trainees. Detailed participant characteristics are presented in Table 4. Incomplete responses were excluded from analysis.

**Table 4:** Baseline characteristics.

Characteristic	Value, *n* (%)
Number of participants	72 (100)
Age	
20-30	16 (22)
30-40	31 (43)
40-50	13 (18)
50-60	7 (10)
60+	5 (7)
Profession	
Board certified neurosurgeon	44 (61)
Neurosurgeon in training	14 (19)
Radiologist	1 (1)
Medical researcher	4 (6)
Researcher	3 (4)
Medical student	6 (8)
Work environment	
Academic hospital	55 (76)
Non-academic public hospital	4 (6)
Private hospital	7 (10)
Research institution	4 (6)
Other	2 (3)
Gender	
Female	19 (26)
Male	53 (74)
Country	
Spain	19 (26)
United States	8 (11)
Italy	6 (8)
Switzerland	11 (15)
Other	28 (39)
Involved in research	65 (90)

Most respondents were aged 30-40 (31/72, 43%). The majority of the respondents work in an academic hospital setting (55/72, 76%), and 65 (90%) reported current involvement in research. The survey participants were mostly male (53/72, 74%). Responses were recruited from multiple countries, with the highest representation from Spain (26%). Complete distribution of the respondents is shown in Table 4.

### 3.2 Results of the survey

#### 3.2.1 Case 1: diagnostic and therapeutic decisions in spinal surgery based on synthetic CT

Confidence in determining (ruling in) the presence of a pars fracture based solely on synthetic imaging was moderate (median 3, IQR 3–4). When respondents were asked how necessary they considered additional conventional imaging if the sCT demonstrated signs of a pars fracture, the respondents found it slightly necessary (median 2, IQR 2-4). Confidence in undergoing surgery without additional conventional imaging was ranked as moderate (median 3, IQR 2-4). When asked to assess the level of confidence in placing lumbar pedicle screws using a navigation system based solely on the synthetic images, respondents reported moderate confidence (median 3, IQR 3-4).

#### 3.2.2 Case 2: diagnostic and therapeutic decisions in hydrocephalus based on sCT

When assessing confidence in diagnosing or excluding hydrocephalus based solely on the synthetic image, respondents reported slight confidence (median 2, IQR 2-3). For the question concerning additional imaging, respondents were asked to consider a hypothetical situation in which the sCT did not show a hydrocephalus. The perceived need to use additional conventional imaging to exclude (ruling out) hydrocephalus safely was high (median 4, IQR 3-4). Respondents reported slight confidence in discharging a patient home based solely on the synthetic image (median 2, IQR 2-3), whereas confidence in placing a navigated external ventricular drain using only the synthetic imaging was moderate (median 3, IQR 2-4).

#### 3.2.3 General attitude towards synthetic imaging

For general diagnostic decision-making (Fig. 3, Table 5), respondents reported a median trust score of 3 (IQR 2-4), reflecting a neutral trust on the 5-point scale. Trust in synthetic images for guiding interventions was also neutral (median 3, IQR 3-4).

**Fig. 3:**
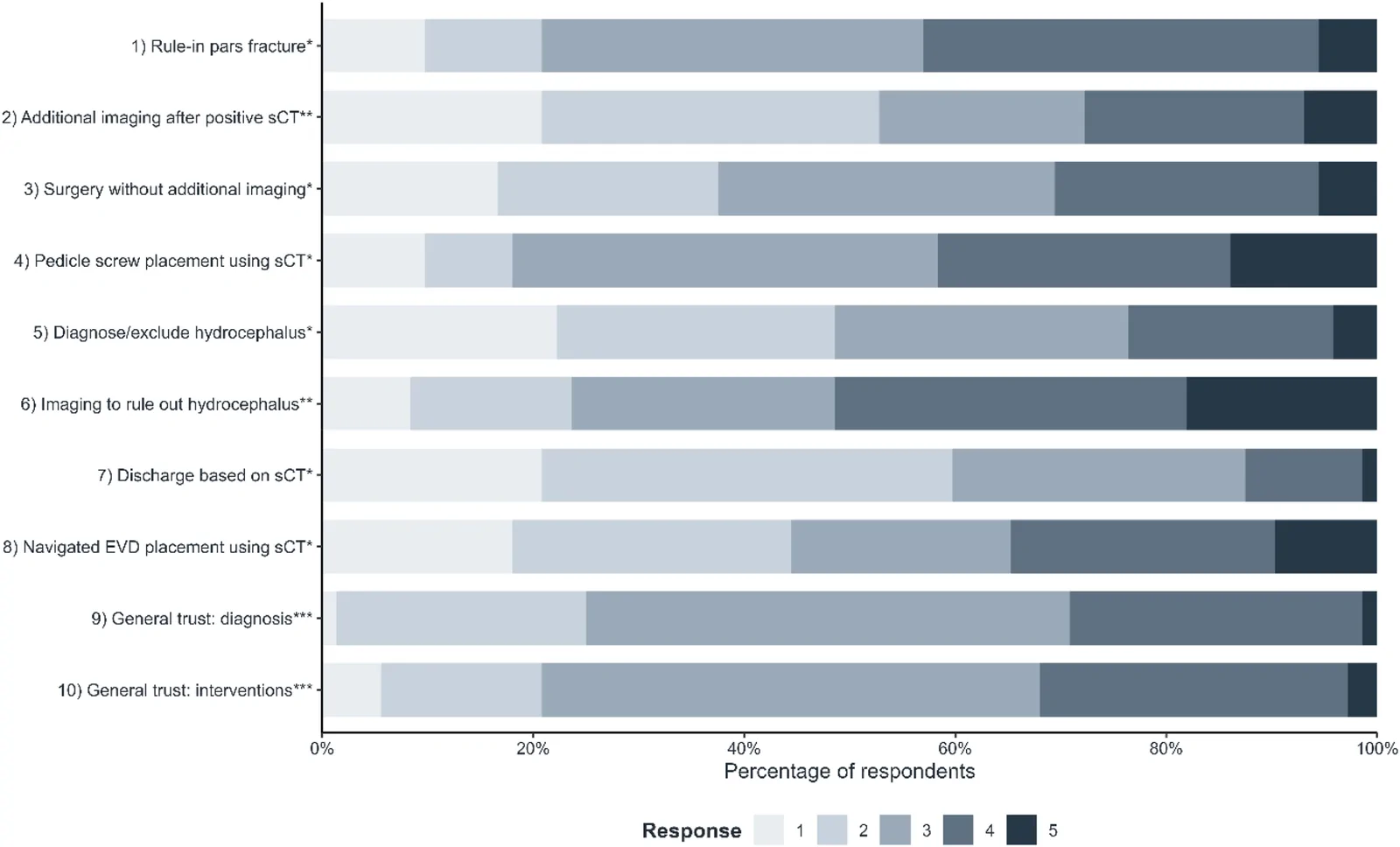
Stacked proportional plot with Likert distribution of attitudes towards synthetic imaging. Stacked proportional plot showing the distribution of attitudes per question. * = Confidence, ** = Necessity, *** = Trust. See Table 7 for detailed questions and explanations of the Likert scale used per attitude.

**Table 5:** Distribution of Likert responses.

Question	1 (n, %)	2 (n, %)	3 (n, %)	4 (n, %)	5 (n, %)
Pars fracture: confidence in diagnosis*	7 (9.7)	8 (11.1)	26 (36.1)	27 (37.5)	4 (5.6)
Pars fracture: need for additional imaging**	15 (20.8)	23 (31.9)	14 (19.4)	15 (20.8)	5 (6.9)
Pars fracture: confidence in surgery without additional imaging*	12 (16.7)	15 (20.8)	23 (31.9)	18 (25)	4 (5.6)
Pars fracture: confidence in pedicle screw placement*	7 (9.7)	6 (8.3)	29 (40.3)	20 (27.8)	10 (13.9)
Hydrocephalus: confidence in diagnosis/exclusion*	16 (22.2)	19 (26.4)	20 (27.8)	14 (19.4)	3 (4.2)
Hydrocephalus: need for additional imaging**	6 (8.3)	11 (15.3)	18 (25)	24 (33.3)	13 (18.1)
Hydrocephalus: confidence in discharge*	15 (20.8)	28 (38.9)	20 (27.8)	8 (11.1)	1 (1.4)
Hydrocephalus: confidence in EVD placement*	13 (18.1)	19 (26.4)	15 (20.8)	18 (25)	7 (9.7)
General attitude: making a diagnosis***	1 (1.4)	17 (23.6)	33 (45.8)	20 (27.8)	1 (1.4)
General attitude: guiding interventions***	4 (5.6)	11 (15.3)	34 (47.2)	21 (29.2)	2 (2.8)

Distributions of the respondents' attitudes per question. * = Confidence, ** = Necessity, *** = Trust. See Table 7 for detailed questions and explanations of the Likert scale used per attitude.

The survey concluded with three multiple-choice questions addressing factors associated with confidence in synthetic imaging (Table 6). Multiple responses were permitted for each question. The most frequently reported factors supporting confidence were the presence of clinical validation studies for specific clinical queries (61/72, 85%; 95% CI [74-92]) and the presence of external validation (50/72, 69%; 95% CI [57-80]). Factors most frequently reported as limiting confidence were poor or non-existent external validation (52/72, 72%; 95% CI [60-82]), no clinical testing for specific clinical queries (45/72, 63%; 95% CI [50-74]), and hallucinations made by generative algorithms (42/72, 58%; 95% CI [46-70]). Finally, they were asked where they see potential benefits for synthetic imaging. More than half of the respondents (47/72, 65%; 95% CI [53-76]) saw benefit for synthetic imaging in low- and middle-income or rural areas with limited access or long waiting times for tomographic imaging. Reducing radiation exposure in patients requiring frequent imaging was identified as an advantage of synthetic imaging by 46 (64%; 95% CI [52-75]) participants. These responses reflect participants' perceived potential benefits and do not represent measurements of clinical effectiveness, cost reduction or improved access.

**Table 6:** Results of general attitudes towards synthetic imaging.

Factors important in building confidence in synthetic imaging applications	Value, n (%)	Factors important in limiting confidence in synthetic imaging applications	Value, n (%)	Where do you see potential benefits for synthetic imaging (e.g. the possibility to generate a moderate-quality synthetic CT from two conventional X-rays)?	Value, n (%)
Variability of diseases present in the training data	46 (64)	Hallucinations made by generative algorithms	42 (58)	Low-middle income or rural areas without easy access to/with very long waiting lists for tomographic imaging	47 (65)
Size of the training dataset	39 (54)	Poor or non-existing external validation	52 (72)	Severely ill patients on the Neuro-ICU who would otherwise require transfer to radiology	38 (53)
Presence of external validation	50 (69)	No clinical testing for specific clinical queries	45 (63)	Pre-hospital situations (i.e. rough initial diagnostics/triage)	40 (56)
Presence of clinical validation studies for specific clinical queries (e.g. rule-in of fractures, rule-out of bleeding)	61 (85)	Algorithms provided for free without involvement of a known MedTech brand	15 (21)	The neurosurgical operating room/guidance of interventions	29 (40)
Peer-reviewed publications	32 (44)			Replacing conventional imaging methods for certain clinical queries	26 (36)
Licensing by a known MedTech brand	13 (18)			Reducing radiation exposure in patients requiring frequent imaging	46 (64)

## 4 Discussion

In this exploratory study, respondents reported different levels of confidence across the clinical scenarios. Overall, confidence was higher for several questions concerning spinal decision-making than for questions concerning cranial applications. Respondents perceived potential benefits of synthetic imaging in settings with limited availability to conventional imaging, such as regions with restricted access. Additionally, reducing radiation exposure in patients requiring frequent imaging was perceived as a potential benefit. These findings highlight areas where synthetic imaging could be most impactful, provided sufficient validation can be established.

Despite the perceived benefits, the results indicate a gap between technical feasibility and confidence to use synthetic imaging for diagnostic and therapeutic decision-making. Although sCT has been proven to be feasible and visually adequate for neurosurgical planning (Staartjes et al., 2021), respondents remained cautious about relying on synthetic imaging for clinical decision-making, particularly in higher-risk situations such as cranial cases. Technical validation alone is therefore not sufficient for clinical adoption.

Notably, respondents reported that clinical validation studies for specific clinical queries and presence of external validation were most important for building confidence in synthetic imaging. Lack of such validation and the risk of hallucinations were frequently selected as factors limiting confidence. These findings indicate that respondents placed importance on evidence demonstrating whether a particular synthetic imaging method can support a certain clinical task, rather than relying solely on general technical descriptions and performance measures obtained during model development.

Confidence in synthetic imaging may also be influenced by other factors such as transparency of model development, perceived uncertainty, and risk evaluation, as suggested by previous research in other clinical contexts (Schulz et al., 2025). Neurosurgeons are required to weigh potential benefits against the risk of rare but severe adverse outcomes on a daily basis. The present survey included clinical decisions with potentially different consequences, such as identifying a suspected pars fracture, excluding hydrocephalus, proceeding with surgery without additional conventional imaging, and discharging a patient solely on synthetic imaging. As mentioned, in synthetic imaging, some voxels are constructed by an algorithm from lower-dimensional input, potentially leading to reconstruction errors. Even when in-silico validation is robust, this distinction may introduce additional uncertainty, especially when the cost of an error is high. Perceived clinical risk may have contributed to respondent's rating, and could explain why respondents show greater acceptance of synthetic imaging in lower-risk scenarios. However, clinical risk perception and the anticipated severity of errors were not directly measured in the present study.

The two algorithms presented differ both in imaging input and reconstruction complexity. Algorithm 1 generated sCT from MRI, converting high-dimensional MRI data into a similar CT representation of the spine. This task is relatively constrained and predictable, which may contribute to higher confidence among respondents. In contrast, Algorithm 2 generates sCT of the skull and brain from only two planar X-ray projections, representing a lower-dimensional input and a more complex reconstruction problem. Consistently lower confidence scores were observed in this cranial scenario, which could suggest that clinicians may perceive greater uncertainty when an algorithm must convert low-dimensional input to high-dimensional output. However, respondents were not directly asked whether information density affected their confidence. Still, these findings could suggest that successful integration of synthetic imaging into neurosurgical practice will require not only technical robustness but also clear communication of uncertainty and risk in clinically meaningful terms. Nevertheless, the two algorithms also differ in various aspects, such as anatomical region, clinical adaptation, pathology type, and validation performance. Therefore, the observed difference should be interpreted cautiously. It is not possible to attribute lower confidence scores solely to the dimensional input and reconstruction problem. Besides, the two algorithms also differed in performance information presented to the respondents. The cranial vignette explicitly stated that not all cases of hydrocephalus were detected. This may partly reflect the observed lower confidence scores in the cranial scenario.

## 5 Limitations

This study has several limitations, due to the survey-based data collection. Participation was voluntary and recruitment was conducted through professional networks and a global database. This strategy may have introduced selection bias, including overrepresentation of respondents with an interest in emerging technologies, medical imaging, or artificial intelligence. The topic of the survey was also complex, which could have had an influence on the response rate. Selective response bias could affect the results by selecting more technically interested/technically native clinicians.

Given the distribution strategy, a precise response rate could not be determined. A number of responses were incomplete or left blank and were therefore excluded from analysis, which could have influenced the representativeness of the data. Reason for incompletion of the survey could not be determined. Also, some participants may not have given fully considered answers because of time constraints. Additionally, data are self-reported and reflect clinicians’ attitudes rather than objective outcomes.

The study population was heterogenous, and the composition of the respondent population limits the extent to which the findings can be generalized to neurosurgeons as a professional group. Although most respondents were board-certified neurosurgeons or neurosurgical trainees, the sample also included respondents from other backgrounds.

To ensure unique participation, each email address could complete the survey only once. This restriction could not be applied to responses submitted via an anonymous survey link, potentially allowing multiple submissions from the same individual. Questions were also presented in a fixed order, which could lead to bias.

Images showed in the survey were not blinded, and therefore directly informed participants which images were real or synthetic. This could introduce assessment bias and may have influenced confidence ratings. We consider the explicit labelling of the synthetic or real image a potential limitation, while noting that it was also intrinsic to the research question of the present survey. Nevertheless, the potential influence of labelling should be considered when interpreting the result.

Besides, each algorithm was represented by a single image pair and a limited number of clinical questions. The selected image pairs represented an average performance by the algorithm. Although this approach reduced respondent burden and allowed the clinical question to be presented in a concise and standardized manner, confidence ratings may have been influenced by the particular image selected, including quality, pathology, and apparent variety between the synthetic and reference images. A single case cannot capture the variability of an algorithm performance.

## 6 Conclusion

In this international survey, confidence in synthetic neuroimaging varied across two clinical scenarios. Respondents frequently identified application-specific validation and external validation performance metrics as important factors supporting confidence, whereas inadequate validation and potential errors were frequently selected as factors limiting confidence.

If these factors are realised, synthetic imaging has the potential to support clinical decision-making, and may for example benefit in areas where access to conventional imaging methods is restricted. Further research should aim to improve validation across different clinical applications and to clearly communicate the reliability of these algorithms to build confidence and support adoption.

## Ethics declaration

The authors have NOT obtained informed consent from participants or their legal representatives. Participation was voluntary and anonymous. Participants were informed about the purpose of the study before starting the questionnaire. Consent was implied by voluntary completion and submission of the survey. No written or verbal consent was obtained.

This study was performed in compliance with relevant laws, regulatory frameworks and guidelines where the research took place. Ethics committee approval was not required under relevant laws and institutional guidelines. No formal ethics committee approval was obtained for this survey, and no formal exemption from ethical review was documented. Participation was voluntary, participants were informed of the purpose of the survey before completing it, and completion of the questionnaire was taken as implied consent.

## Contributors

Michelle D. Poelman designed the survey, collected and analysed the data, and wrote the main manuscript. Victor E. Staartjes supervised the study, contributed to the survey design and manuscript writing, and provided critical revisions. Marc L. Schröder contributed to manuscript writing and provided critical revisions. Olivier Zanier, Istiak Khan, Daniel de Wilde, Stefanos Voglis, Adrian Elmi-Terander, Victor Gabriel El-Hajj, Nikolay Gabrovsky, Alessandro Boaro, Stefan Motov, Eric Suero Molina, Seung-Jun Ryu, Antonio Di Ieva, Marisa Gandia González, Luca Regli, Carlo Serra reviewed the manuscript and provided critical revisions. This survey was supported by the EANS Emerging Technologies and Innovations in Neurosurgery (ETIN) taskforce. All authors reviewed the manuscript and approved the final version.

## Data availability

Data is available from the corresponding author upon reasonable request.

## Funding

No funding was received for this research.

## Competing interest

The authors declare no competing interests.
