# Supplementary material for: Clinicians’ confidence in synthetic neuroimaging: a survey from the EANS emerging Technologies and Innovations in Neurosurgery (ETIN) taskforce
Source: Brain Spine. 2026 Oct 22;6:107488. doi: 10.1016/j.bas.2026.107488 (PMC13718392; doi:10.1016/j.bas.2026.107488)
Supplement: Supplementary file 1 [file mmc1.docx]

**Supplement**

**Table 7**

*All questions from the survey*

| **Question Type** | **Content** | **Response options** | **Response type** |
| --- | --- | --- | --- |
| Introduction | Thank you very much for taking the time to participate in our survey. Synthetic imaging methods, such as image modality conversion and synthetic tomography, are currently being developed and validated and have clear potential to aid neurosurgical patients. However, any clinical implementation would require a certain level of trust from both clinicians and patients regarding these synthetic images. The aim of this study is to assess the current attitude of neurosurgeons toward synthetically generated images of the brain and spine for diagnostic and therapeutic purposes.   Thank you for your time, Michelle Poelman Victor Staartjes Marisa Gandia González | - | Information |
| General information | Please select your age group | 1. 20-30 2. 30-40 3. 40-50 4. 50-60 5. 60+ | Single choice |
| General information | Please select your profession | 1. Board certified neurosurgeon 2. Neurosurgeon in training 3. Radiologist 4. Medical researcher 5. Medical student 6. Other | Single choice |
| General information | What environment do you primarily work in? | 1. Academic hospital 2. Non-academic public hospital 3. Private hospital 4. Research institution 5. Other | Single choice |
| General information | Please select your gender | 1. Male 2. Female 3. Non-binary / third gender 4. Prefer not to say 5. Other | Single choice |
| General information | Which country are you primarily located in? | All countries | Single choice |
| General information | Do you currently participate in medical research? | Yes / No | Single choice |
| Information | In the following sections we will describe several different algorithms along with their level of validation and an example input and output. Then we will ask you to rate whether your level of trust in these algorithms is sufficient to make a diagnostic decision. Some of the presented algorithms are real, published and validated, while some are fictitious or simply not yet published.  Please rate your confidence in the produced images on a Likert scale: 1: Not confident at all 2: Slightly confident 3: Moderately confident 4: Highly confident 5: Completely confident | - | Information |
| Algorithm 1 | **Algorithm 1: Synthetic CT from MRI** This algorithm is based on an MRI sequence that adds a couple of minutes to a normal MRI and teases out some information about the bone structure. Then, a convolutional neural network (CNN) transforms the MRI to a synthetic CT (sCT). The algorithm has been trained on hundreds of paired data sets and it has been externally validated. The same technique has also been succesfully applied to other joints/bones. The training data set included several patients with pars fractures.  Below is an example of the generated sCT alongside the actual spiral CT. (**Figure 1**) | - | Information |
| Algorithm 1  Synthetic imaging for **diagnostic** purposes | Based on the information provided above and on the image quality you have been able to assess from the images above:  How confident are you in determining (ruling in) the presence of a pars fracture based solely on the synthetic image? | 1: Not confident at all 2: Slightly confident 3: Moderately confident 4: Highly confident 5: Completely confident | Single choice |
| Algorithm 1  Synthetic imaging for **diagnostic** purposes | Based on (…) images above:  If the sCT shows signs of a pars fracture, how necessary would you deem additional conventional imaging to confirm the diagnosis? | 1: Not necessary at all 2: Slightly necessary 3: Moderately necessary 4: Very necessary 5: Absolutely necessary | Single choice |
| Algorithm 1  Synthetic imaging for **diagnostic** purposes | Based on (…) images above:  If there were an indication for surgery, how confident would you be in undergoing surgery without additional conventional imaging? | 1: Not confident at all 2: Slightly confident 3: Moderately confident 4: Highly confident 5: Completely confident | Single choice |
| Algorithm 1  Synthetic imaging for **therapeutic** purposes | Based on (…) images above:  How confident would you be in placing lumbar pedicle screws using a navigation system based solely on the synthetic images (sCT) without additional imaging? | 1: Not confident at all 2: Slightly confident 3: Moderately confident 4: Highly confident 5: Completely confident | Single choice |
| Algorithm 2 | **Algorithm 2: Synthetic CT of biplanar X-ray**  This algorithm is based on a generative adversarial network (GAN) that can synthesize a tomography-like image from a.p. and lateral X-rays of the skull and brain. The algorithm has been developed on hundreds of paired data sets. It has been externally validated. During external validation, the algorithm has been able to pick up most but not all of the hydrocephalus cases. The training data set included several patients with hydrocephalus.  Below is an example of the actual spiral CT alongside the generated sCT. (**Figure 2**) | - | Information |
| Algorithm 2  Synthetic imaging for **diagnostic** purposes | Based on the information provided above and on the image quality you have been able to assess from the images above:  How confident are you in diagnosing or excluding a hydrocephalus based solely on the synthetic image? | 1: Not confident at all 2: Slightly confident 3: Moderately confident 4: Highly confident 5: Completely confident | Single choice |
| Algorithm 2  Synthetic imaging for **diagnostic** purposes | Based on (…) images above:  If the sCT does not show a hydrocephalus, how necessary do you deem additional conventional imaging to safely exclude (ruling out) a possible hydrocephalus? | 1: Not necessary at all 2: Slightly necessary 3: Moderately necessary 4: Very necessary 5: Absolutely necessary | Single choice |
| Algorithm 2  Synthetic imaging for **diagnostic** purposes | Based on (…) images above:  How confident would you be in discharging the patient home based only on the synthetic image? | 1: Not confident at all 2: Slightly confident 3: Moderately confident 4: Highly confident 5: Completely confident | Single choice |
| Algorithm 2  Synthetic imaging for **therapeutic** purposes | How confident would you be in placing a navigated external ventricular drain based on a navigation system based only on the synthetic imaging? | 1: Not confident at all 2: Slightly confident 3: Moderately confident 4: Highly confident 5: Completely confident | Single choice |
| General attitude | In the following section, we will ask questions about your general trust in synthetic imaging. Please rate your confidence in the synthetic images on a Likert scale: 1: No trust at all 2: Low trust 3: Neutral trust 4: High trust 5: Complete trust | - | Information |
| General attitude | In general, to what extent would you trust synthetic images (images that are partially generated by algorithms, rather than every voxel/pixel being based on a physical measurement) for **making a diagnosis**? | 1: No trust at all 2: Low trust 3: Neutral trust 4: High trust 5: Complete trust | Single choice |
| General attitude | In general, to what extent would you trust synthetic images (images that are partially generated by algorithms, rather than every voxel/pixel being based on a physical measurement) for **guiding interventions**? | 1: No trust at all 2: Low trust 3: Neutral trust 4: High trust 5: Complete trust | Single choice |
| General attitude | Factors important to you in building confidence in synthetic imaging applications | 1. Variability of diseases present in the training data 2. Size of the training data set 3. Presence of external validation 4. Presence of clinical validation studies for specific clinical queries (e.g. rule-in of fractures, rule-out of bleeding) 5. Peer-reviewed publications 6. Licensing by a known MedTech brand | Multiple choice, more answers allowed |
| General attitude | Factors important to you in limiting confidence in synthetic imaging applications: | 1. Hallucinations made by generative algorithms 2. Poor or non-existing external validation 3. No clinical testing for specific clinical queries 4. Algorithms provided for free without involvement of a known MedTech brand | Multiple choice, more answers allowed |
| General attitude | Where do you see potential benefits for synthetic imaging (e.g. the possibility to generate a moderate-quality synthetic CT from two conventional X-rays)? | 1. Low-middle income or rural areas without easy access to / with very long waiting lists for tomographic imaging 2. Severely ill patients on the Neuro-ICU who would otherwise require transfer to radiology 3. Pre-hospital situations (i.e. rough initial diagnostics / triage) 4. The neurosurgical operating room / guidance of interventions 5. Replacing conventional imaging methods for certain clinical queries 6. Reducing radiation exposure in patients requiring frequent imaging | Multiple choice, more answers allowed |
